# Psychosocial and Behavioral Correlates of Sleep Duration and Sleep Disturbance Among Lesbian, Gay, and Bisexual Women

**DOI:** 10.3390/ijerph23020201

**Published:** 2026-02-04

**Authors:** Jennifer M. Jabson Tree, Katherine Buchman

**Affiliations:** Department of Public Health, Purdue University, West Lafayette, IN 47906, USA; kbuchman@purdue.edu

**Keywords:** sleep disturbance, sleep duration, psychosocial characteristics, sexual orientation

## Abstract

**Highlights:**

**Public health relevance—How does this work relate to a public health issue?**

- Lesbian, gay, and bisexual women experience inequities in sleep duration and disturbance when compared to heterosexual women, and solutions to improve this may include understanding the psychosocial factors that can be added to evidence-based behavioral interventions to improve sleep for LGB women. However, the most relevant psychosocial factors in sleep for LGB women have not yet been documented.
- Psychosocial characteristics were significantly associated with sleep outcomes for LGB women. Social strain and negative emotional expressiveness were negatively associated with sleep outcomes, while social function and optimism were positively associated with sleep outcomes. Health behaviors, including current smoking, alcohol consumption, and exercise, were not significantly associated with sleep outcomes for LGB women.

**Public health significance—Why is this work of significance to public health?**

- Sleep disturbance, insomnia, and shortened sleep as experienced by LGB women may be improved by implementing evidence-based interventions that include or address social strain, negative emotional expressiveness, and optimism. This is significant because sleep is a central pillar in women’s health, where quality sleep outcomes are associated with improvements in anxiety and depression as well as chronic disease—disease states that LGB women experience disproportionately higher than heterosexual women.

**Public health implications—What are the key implications or messages for practitioners, policy makers, and/or researchers in public health?**

- Many sleep experts focus on health behaviors (e.g., tobacco use, alcohol, physical activity) to address sleep problems. Our findings suggest that this is an evidence-based approach for heterosexual women; however, among LGB women, psychosocial factors may be more relevant to sleep outcomes. Practitioners could consider sexual orientation and psychosocial experiences when engaging in solutions to address sleep. Researchers could begin to explore how to incorporate social strain, negative emotional expressiveness, social function, and optimism into evidence-based interventions for sleep improvement among LGB women.

**Abstract:**

Introduction: Sleep is an essential dimension of good physical and mental health. Lesbian, gay, and bisexual (LGB) women experience inequities in sleep duration and disturbance compared to heterosexual women. Psychosocial and behavioral characteristics are important to sleep in the general population; they may advance our understanding about sleep inequities among LGB women and provide key information for developing promising interventions. Methods: Data for this project were provided by the Women’s Health Initiative (WHI). The sample size for this project was 1436: 884 LG women and 552 bisexual women. Outcome variables were sleep duration and disturbance. The authors sought to clarify the associations, including the strength, between psychosocial factors and sleep outcomes among LGB women. Associations between psychosocial characteristics, health behaviors, and sleep outcomes were tested using multivariable, hierarchical, nested, linear regression models, stratified by sexual orientation. Results: Social strain, social function, optimism, and negative emotional expressiveness were significantly associated with sleep outcomes for LGB women. Health behaviors were not consistently or strongly associated with sleep outcomes for LGB women. Conclusions: The findings point to the importance of social strain, social function, negative emotional expressiveness, and optimism in LGB women’s sleep. It is possible that LGB women’s sleep could be improved with evidence-based interventions that use our findings.

## 1. Background

The National Sleep Foundation (NSF) [1] and the Centers for Disease Control and Prevention (CDC) [2] recommend that adults should get seven to nine hours of sleep nightly. However, according to the CDC 40% of adults are getting insufficient sleep [2]. Sleep problems, including insomnia, poor sleep, shortened sleep duration, and other forms of sleep disruption, negatively impact the immune system, quality of life, mood, cognitive functioning, physical health, and risks for disease and injury, including workplace and motor vehicle accidents [1,3,4]. Sleep problems and insufficient sleep have been linked with diabetes, obesity, cardiovascular disease, and metabolic syndrome [4].

### 1.1. Sleep Inequities and LGB Women

There is a growing body of empirical evidence documenting that marginalized groups [5], including lesbian, gay, and bisexual (LGB) women, experience worse sleep in the form of greater sleep disturbance and shorter sleep duration than other groups. Specifically, from self-report measures of sleep duration and quality, lesbian and gay (LG) women report worse sleep than heterosexual women [6,7,8], heterosexual men, and gay men. Self-report measures of sleep quality and duration for bisexual women also indicate more sleep problems [6,7,8,9] and shorter sleep duration than LG and heterosexual women [6,9].

There is nothing inherent to LGB women’s biology that would cause poor sleep. It is theorized that chronic exposure to minority stressors [10] in the form of persistent anti-LGB legislation [11,12] and policies results in LGB women experiencing constant/continuous/regular discrimination, marginalization, and threats of violence [13]. These chronic stressors may contribute to sleep disturbances and shorter sleep duration. An estimated 65% of LGB adults experience discrimination [14]. Extrapolating from evidence involving other minoritized populations, discrimination is negatively associated with sleep outcomes [15,16,17]. It is possible that discrimination experienced by LGB groups may also result in sleep problems [18]. LGB women experience disparities in several health outcomes that in other populations have been associated with sleep, including obesity, being overweight [19,20,21], anxiety, and depression [21]. LGB women’s sleep is understudied. By evaluating LGB women’s sleep, we could provide a next step in our understanding of LGB women’s health inequities.

### 1.2. Theory

The Health Equity Promotion Model (HEPM) was used to guide our evaluation and description of psychosocial and behavioral correlates of sleep disturbance and sleep duration among LGB women. According to the HEPM [22], health outcomes, including sleep outcomes, are influenced by two categories of variables, including health-promoting and -adverse pathways. This includes behavioral, social and community, psychological, and biological pathways. Following the HEPM, our study tested for direct associations between health-promoting and -adverse pathways made up of psychosocial (psychological and social) and behavioral factors and sleep outcomes among LGB women.

### 1.3. Psychosocial Factors and Sleep

In this project we refer to psychological and social factors as psychosocial factors-- variables that represent how someone feels about themselves in relation to social context and resources [23]. Psychosocial factors known to successfully predict LGB women’s health [23,24] and posited to be associated with sleep [25,26,27] are included: social strain, social function, social support, optimism, emotional expressiveness, and hostility. There is also a growing interest in how these psychosocial factors may be associated with sleep quality [28]. Aggarwall et al. [28] found that low levels of social support were associated with a 78% greater likelihood of insomnia in women (aOR: 1.78, 95%CI = 1.18–2.71). Extremely limited empirical evidence exists about how psychosocial factors relate to sleep among LGB women. Understanding more about the relationship between these psychosocial factors and sleep among LGB women could facilitate the development of efficacious interventions to assist in improving sleep outcomes as well as other disparities.

### 1.4. Health Behaviors

Several health behaviors, including smoking, alcohol use, and physical activity, may be important correlates of women’s sleep and could point to promising intervention mechanisms for improving sleep. Among women, smoking tobacco disrupts sleep architecture and therefore diminishes sleep quality [29,30,31,32]. Inkelis et al.’s [30] systematic review demonstrates that due to its neurotoxic nature, alcohol consumption consistently and negatively impacts sleep problems, causing frequent sleep disruptions and awakenings among women [33,34]. Evidence concerning physical activity and sleep quality and duration among women is mixed; some studies suggest that engaging in physical activity improves sleep outcomes and others report that physical activity does not improve sleep quality and duration. Unfortunately, none of the aforementioned studies or reviews analyzed outcomes according to sexual orientation.

Empirical evidence is desperately needed concerning associations between psychosocial factors, health behaviors, and sleep duration among LGB women. Evaluating and documenting these associations could provide necessary data to inform future data-driven and evidence-based sleep interventions for LGB women, and it may provide necessary evidence to improve the capacity for sleep-health providers to meet the needs of LGB patients. Without such evidence, it is probable that interventions and policy, practices, and procedures will continue to exclude LGB women.

## 2. Methods

### 2.1. Study Sample

This study used data from the Women’s Health Initiative (WHI); WHI design, methodology, and sampling strategies have been described in great detail in previous publications [35]. Beginning in 1993, the WHI recruited post-menopausal women aged 50–79 into one of two study arms: an observational trial or the longitudinal clinical trial. Data from both arms were included in the present study. The sample size for this project was 1436: 884 LGB women and 552 bisexual women. We excluded cases missing data specific to sexual orientation, sleep quality, and psychosocial items.

WHI data were collected more than 10 years ago. However, the importance of psychosocial variables in the health of LGB women persists and the social context including sexual-orientation related discrimination and stigma faced by LGB women has been minimally improved since the WHI data were collected [14,36]. For example, in 2025, 575 anti-LGBT bills were active in the US, many including barriers to healthcare [12]. Therefore, these data are still useful in understanding the psychosocial correlates of sleep among LGB women.

This study comprised secondary data analysis and was approved by the Purdue University IRB board (protocol #2024-64).

### 2.2. Measures and Variables

At the time the data were collected, only sexual behavior during older adulthood, self-reported by participants, was used to define sexual orientation. This analysis used the same item to assess sexual orientation as has been used in other publications using WHI data [37,38]: “Regardless of whether you are currently sexually active, which response best describes who you have had sex with over your adult lifetime.” Possible responses included “have never had sex,” “sex with a woman or with women,” “sex with a man or with men,” “sex with both men and women,” and “prefer not to answer.” Participants who indicated that they had had sex with only women (lesbian/gay) or both women and men (bisexual) were included in our analysis.

Sleep duration was assessed with one item, as has been published by others using WHI data [3]: “About how many hours of sleep did you get on a typical night during the past 4 weeks?”. Response options were “5 or less hours,” “6 h,” “7 h,” “8 h,” “9 h,” or “10 or more hours.” Response options were scored low to high, where “5 or less hours” equaled 1 and “10 or more hours” equaled 6. Short sleep was defined as ≤6 h/day of total sleep [1,39]. Women who reported sleeping ≥7 to 9 h/day were considered to be sleeping the recommended amounts, and women sleeping ≥10 h/day were considered long sleepers [39].

Sleep disturbance was measured as it has been in other publications using WHI data [3], with a composite scale of 5 items; only participants who answered all 5 items were included. Example questions included “Did you have trouble falling asleep?”, with answer options “No, not in past 4 weeks” (0) and “Yes, less than once a week” (1). For the question, “Did you wake up several times per night?”, response options were (1) “Yes, 1 or 2 times a week,” (2) “Yes, 3 or 4 times a week,” and (3) “Yes, 5 or more times a week.” Sleep disturbance scores ranged from 0 to 20, with higher scores indicating greater sleep disturbance.

Social function [40] was determined by averaging 2 items, with a range of 0 to 100, where higher scores indicated better social function. The items asked participants how much time in the last month social activities were interrupted by physical health and emotional problems.

Social strain [41] was determined through a combined score of 4 items and is defined as a marker of “negative social support.” Higher scores on the combined scale were indicative of higher social strain; scores ranged from 4 to 20.

Social support [42] was determined through a combined score of 9 items, where higher scores were indicative of greater social support, and the scores ranged from 9 to 45. Social support was defined as the amount of social support reported by participants and was broken into various types of social support as described by those 9 items.

Optimism [43] was measured with six questions, with the score ranging from 6 to 30, where higher scores indicated greater levels of optimism and lower scores indicated greater pessimism. Optimism was defined as the amount of optimism reported by participants. Negative emotional expressiveness [44] was measured with three items, and the total score ranged from one to five, where five indicated greater negative emotional expressiveness. The items were from the Ambivalence over Emotional Expressiveness questionnaire. Hostility was measured with thirteen true/false items that were summed and ranged from 0–13, where a higher score indicated greater hostility. The items are from the cynicism subscale from the Cooke–Medley questionnaire.

Health behaviors were assessed at baseline by asking participants if they were current smokers (yes 1/no 0), the number of servings of alcohol consumed weekly, and the number of minutes of recreational physical activity per week.

Participants self-reported race/ethnicity, highest level of education completed, and income, as described by others [35].

### 2.3. Statistical Analyses

Descriptive and summary statistics were calculated for outcome, psychosocial, health behavior, and demographic variables. Multivariable hierarchical, three-block, linear regressions were calculated to test for associations between psychosocial characteristics, health behaviors, and sleep outcomes, by sexual orientation: lesbian/gay and bisexual women. Using hierarchical multiple linear regression was intentional, to uncover the possible existence of (1) associations between psychosocial factors and sleep outcomes, and health behaviors and sleep outcomes, across sexual orientations and (2) to describe how associations between psychosocial factors and sleep outcomes changed or remained constant after health behavior variables were added to the hierarchical linear regression models across sexual orientation groups. All multivariable, hierarchical regression models were adjusted for income, education, and race/ethnicity (block 1). Standardized beta coefficients are reported for each multi-variable model, given that items were measured on multiple different scales. All analysis for this study was completed using StataNow software, version 18.5 [45].

## 3. Results

Descriptive and summary statistics for the sample, by sexual orientation, are summarized in Table 1. There were statistically significant demographic differences between lesbian/gay and bisexual women, including education (x^2^ = 19.94, *p* = 0.001) and income (x^2^ = 24.74, *p* = 0.002). The sexual orientation groups were very similar among the outcome measures; sleep duration (x^2^ = 29.49; *p* = 0.08) appeared to be trending toward statistical significance by sexual orientation.

### 3.1. Psychosocial Factors, Health Behaviors, and Sleep Disturbance, by Sexual Orientation

#### 3.1.1. Lesbian/Gay Sleep Disturbance and Duration

Social strain, social function, and optimism were associated with sleep disturbance in the anticipated directions among LGB women in model one (Table 2). As social function (β = −0.23, *p* < 0.001) and optimism (β = −0.13, *p* = 0.001) increased, sleep disturbance decreased. As social strain increased (β = 0.10, *p* = 0.01), sleep disturbance also increased.

Model two included added health behaviors. Only recreational physical activity (β = −0.09, *p* = 0.03) was associated with sleep disturbance; as minutes of recreational physical activity increased, sleep disturbances decreased. Psychosocial characteristics that were significant in model one remained significant in model 2; social strain (β = 0.11, *p* = 0.02), social function (β = −0.05, *p* < 0.001), and optimism (β = −0.11, *p* = 0.02) were all significantly associated with sleep disturbance in the directions observed in model one.

Social strain, social function, optimism, negative emotional expressiveness, and hostility were associated with sleep duration in the anticipated direction among lesbian/gay women in model one (Table 3). As social strain (β = −0.07, *p* = 0.05) and negative emotional expressiveness (β = −0.08, *p* = 0.03) increased, sleep duration decreased. As social function (β = 0.09 *p* = 0.01), optimism (β = 0.01, *p* = 0.01), and hostility (β = 0.09, *p* = 0.02) increased, sleep duration increased.

Model two included added health behaviors. None of the health behaviors were associated with sleep duration among LGB women. Psychosocial characteristics remained significant as in model one; social function (β = 0.11, *p* = 0.02), negative emotional expressiveness (β = −0.1, *p* = 0.02), and hostility (β = 0.12, *p* = 0.02) were all significantly associated with sleep duration in the directions observed in model one.

#### 3.1.2. Bisexual Sleep Disturbance and Duration

Social strain and social function were associated with sleep disturbance in the anticipated direction among bisexual women in model one (Table 2). As social strain (β = −0.12, *p* = 0.02) increased, sleep duration decreased. As social function (β = −0.17 *p* < 0.001) increased, sleep disturbance increased.

Model two included added health behaviors. None of the health behaviors were significantly associated with sleep disturbance. Among the psychosocial characteristics, social function (β = −0.13, *p* = 0.04) remained significant and in the directions observed in model one.

Social strain was associated with sleep duration in the anticipated direction among bisexual women in model one (Table 3). As social strain (β = −0.1, *p* = 0.04) increased, sleep duration decreased.

Model two included added health behaviors. None of the health behaviors were associated with sleep duration. Social strain (β = −0.13, *p* = 0.05) remained significantly associated with sleep duration and in the anticipated direction.

## 4. Discussion

The Health Equity Promotion Model (HEPM) [46] was used to guide our description and evaluation of the psychosocial and behavioral correlates of sleep disturbance and sleep duration among LGB women. Our analyses revealed that for LGB women, psychosocial characteristics, including optimism, social strain, social support, negative emotional expressiveness, and hostility, were strongly associated with sleep duration and disturbance, but health behaviors were not associated with sleep quality.

The existing evidence concerning sleep quality and the psychosocial and behavioral correlates of sleep among LGB women is sparse and incomplete. Therefore, we drew from both the published evidence concerning the psychosocial correlates of sleep among women “in general” and the evidence documenting sleep among other marginalized populations.

Our findings concerning optimism and sleep are consistent with those published by others. Women in the general population who feel positive about their lives and report expecting good things also report better sleep duration and fewer sleep disturbances [47,48,49]. Similarly, we found that as optimism increased, sleep duration increased and sleep disturbances decreased among LG women. This aligns with HEPM, which associates optimism with the “psychological” or “social and community” health-promoting pathways. However, this similarity was not found for bisexual women. We could not determine from this dataset whether these individuals currently had male or female partners nor for what duration they had been with partners of either sex. It could be that findings differ for bisexual women with primarily female partners versus those who had primarily male partners. This may be an area for future research endeavors.

Although there is very little evidence about social support and sleep among LGB groups, we can extrapolate from other, albeit minimal, existing studies concerning other marginalized groups. The limited available publications indicate that among both the general population and other (not LGB) marginalized groups who may face systemic discrimination and stigma, social support is positively associated with sleep quality and duration. In both the general population and in some marginalized groups, social support is associated with sleep quality and duration [26,50,51,52]. In studies focused on sleep duration among Black and Brown people, social support was also associated with sleep duration. Specifically, lower social support ratings were associated with shorter sleep duration [50]. However, in our sample of LGB women, social support was not associated with either sleep duration or disturbance.

Perhaps we failed to find an association between social support and sleep because the individuals who fulfill these types of social supports to LGB women also expose them to social stigma and discrimination. This is supported by the HEPM, which identifies these types of experiences as having the capacity to be health-promoting or -adverse pathways. For example, if LGB women received social support from family in the form of childcare assistance (as assessed by the MOS item), but the family members do not affirm LGB women or microaggress them because of their sexual orientation, it is possible that any benefit produced by social support could be negated.

Studies that find social strain to be associated with sleep outcomes speculate that social strain may affect sleep through other psychosocial variables, such as loneliness [25]. However, most samples in the published literature have not considered social strain experienced by mid-life LGB populations. One possible explanation for the association we identified between social strain and sleep in our sample involves minority stressors and exposure to chronic discrimination caused by anti-LGBT social norms, policies, and laws [10,53]. Slopen and colleagues [17] found a consistent association between experiences of discrimination and sleep outcomes, where discrimination was associated with poorer sleep outcomes. Perhaps the association between social strain and sleep is mediated by toxic, systemic, minority stressors that exist beyond any individual’s capacity for change. This aligns with the importance that the HEPM places on social stressors such as stigma and exclusion, as a structural-level element.

Negative emotional affect is positively associated with sleep efficiency and global sleep indices [27]; however, few studies have investigated the associations between negative emotional expressiveness [44] and sleep or associations with sleep among LGB women. We found that negative emotional expressiveness was negatively associated with sleep duration among LG women only. Ours is a cross-sectional data source, so it is impossible to know if negative emotional expressiveness caused shorter sleep duration or if shorter sleep duration caused diminished emotional expressiveness.

Hostility has been shown to be negatively associated with several health problems, including increased illness, cardiovascular diseases and all-cause mortality [54,55]. Researchers suggest that there are three models that explain this relationship. The behavioral model suggests that individuals scoring higher on hostility scales may be more likely to engage in health-diminishing behaviors such as alcohol consumption and tobacco use. The psycho-physiological reactivity model suggests that hostility may create risks to health through sympathetic nervous system reactions. The psychosocial vulnerability model suggests that individuals scoring high in hostility may experience more stressful life events, have smaller social networks, and receive less social support that could counteract the negative consequences of hostility [56]. Several studies have shown that individuals with high levels of hostility experience poorer sleep than controls [51,52,53]. However, we found a positive association between hostility and sleep duration among LG women. This may be an unanticipated artifact of this sample or may represent an important new finding specific to LG women. Additional research is needed to clarify this finding.

### 4.1. Limitations

Although WHI is a longitudinal study, the data available for this project were cross-sectional, precluding us from assessing the causal nature of the independent and dependent variables. Objective measures of affect and sleep were not available. The data used in this study were collected during the baseline data collection for the WHI. Finally, measures of sexual orientation and gender in this dataset were not aligned with current best practices for assessing sexual orientation and gender identity [57,58].

### 4.2. Strengths

WHI is among the first long-term studies of women’s health that asked questions concerning their sexual orientation [24]. This has allowed us to understand new and emerging questions about psychosocial factors and sleep duration and quality among LGB women. These data also include one of the most complete assessments of psychosocial characteristics in any large health surveillance effort for women’s health. This has facilitated our ongoing effort to understand and investigate the relationships between psychosocial characteristics and women’s health and sleep.

## 5. Conclusions

Sleep inequities identified by sexual orientation do not exist due to genetic or biologically inherent problems in LGB women. Rather, it is likely the lived experience of being LGB in a staunchly heteronormative world and the resulting exposures to discrimination interpersonally, institutionally, and structurally that cause sleep inequities. Our findings point to the importance of social strain, social function, negative emotional expressiveness, and optimism in LGB women’s sleep. Until we can ensure comprehensive and consistent protections against anti-LGBTQ+ policies, laws, social norms, and expectations, evidence-based interventions should be implemented that support LGB women’s sleep by coping with multi-level sources of discrimination and stigma. However, both activities are needed to fully address sleep inequities and their associated health outcomes.

## Figures and Tables

**Table 1:** Descriptive characteristics of the sample.

Sample Characteristics		LG-Women (*n* = 884)	Bi-Women (*n* = 552)		
		*n*/Mean (%/*SD*)		*t*/*χ*^2^/*F*	*p*
Outcomes	Sleep Disturbance	869/7.01 (4.52)	538/7.17 (4.71)	29.49	0.08
	Hours of Sleep	2.89 (1.05)	2.91 (1.06)	3.12	0.68
Psychosocial Characteristics	Social Functioning (0–100)	878/85.04 (21.39)	545/82.89 (23.51)	13.03	0.11
	Social Strain	875/6.68 (2.43)	537/6.77 (2.61)	15.73	0.20
	Social Support	871/36.89 (7.46)	530/35.49 (7.52)	62.11	0.002
	Optimism	876/23.88 (3.488)	538/23.46 (3.90)	35.08	0.03
	Negative Emotional Expressiveness	879/2.90 (0.62)	541/2.91 (0.62)	20.44	0.16
	Hostility	862/3.28 (2.73)	529/3.92 (2.75)	29.71	0.005
Health Behaviors	Smoking Now (% yes)	575/0.14 (0.35)	343/0.10 (0.30)	3.37	0.061
	Alcoholic Drinks Weekly	578/2.89 (5.36)	343/3.19 (5.23)	−0.84	0.39
	Minutes of Recreational Physical Activity Weekly	578/199.92 (195.73)	343/186.33 (182.48)	1.04	0.30
Demographic Characteristics	**Race/Ethnicity**			9.84	0.08
	American Indian/Alaska Native	2 (0.23)	3 (0.55)		
	Asian or Pacific Islander	5 (0.57)	10 (1.82)		
	Black or African American	59 (6.67)	45 (8.21)		
	Hispanic/Latino/a	26 (2.94)	17 (3.10)		
	White	777 (87.90)	469 (85.58)		
	Other	15 (1.70)	4 (0.73)		
	**Education**			19.94	0.001
	Less than high school diploma	18 (2.06)	14 (2.55)		
	High School Diploma	38 (4.35)	43 (7.85)		
	Some college/vocational school	207 (23.71)	151 (27.55)		
	College Graduate	73 (8.36)	60 (10.95)		
	Post Graduate	138 (15.61)	84(15.33)		
	Graduate Degree	399 (45.70)	196 (35.77)		
	**Income**			24.74	0.002
	Less than $10,000	30 (3.46)	37 (6.80)		
	$10,000 to $19,999	68 (7.83)	65 (11.95)		
	$20,000 to $34,999	191 (22.00)	133 (24.45)		
	$35,000 to $49,999	175 (20.16)	97 (17.83)		
	$50,000 to $74,999	209 (24.08)	102 (18.75)		
	$75,000 to $99,999	96 (11.06)	59 (10.85)		
	$100,000 to $149,999	66 (7.60)	38 (6.99)		
	$150,000 or more	24 (2.76)	6 (1.10)		
	Don’t know	9 (1.02)	7 (1.29)		

**Table 2:** Psychosocial correlates of sleep disturbances among LGB women.

LG Women Sleep Disturbances					
		B	SE	*b*	*p*
Model 1					
Psychosocial Characteristics	Social Strain	0.2	0.07	0.1	0.01
	Social Function	−0.05	0.01	−0.23	<0.001
	Social Support	−0.03	0.02	−0.06	0.14
	Optimism	−0.17	0.05	−0.13	0.001
	Negative Emotional Expressiveness	0.02	0.25	0.003	0.94
	Hostility	−0.11	0.06	−0.07	0.07
Model 2					
Psychosocial Characteristics	Social Strain	0.21	0.09	0.11	0.02
	Social Function	−0.05	0.01	−0.24	<0.001
	Social Support	−0.01	0.03	−0.02	0.64
	Optimism	−0.15	0.07	−0.11	0.02
	Negative Emotional Expressiveness	0.04	0.31	0.01	0.9
	Hostility	−0.13	0.08	−0.08	0.09
Health Behaviors	Smoking	−0.32	0.55	−0.02	0.56
	Alcohol Consumption	0.05	0.03	0.06	0.16
	Recreational Physical Activity	−0.002	0.001	−0.09	0.03
	Bisexual Women Sleep Disturbance				
		B	SE	*b*	*p*
Model 1					
Psychosocial Characteristics	Social Strain	0.2	0.09	0.12	0.02
	Social Function	−0.03	0.01	−0.17	<0.001
	Social Support	0.05	0.03	0.07	0.15
	Optimism	−0.11	0.06	−0.1	0.06
	Negative Emotional Expressiveness	0.41	0.36	0.05	0.25
	Hostility	−0.09	0.09	−0.06	0.29
Model 2					
Psychosocial Characteristics	Social Strain	0.2	0.11	0.12	0.08
	Social Function	−0.02	0.01	−0.13	0.04
	Social Support	−0.01	0.04	−0.01	0.89
	Optimism	−0.05	0.08	−0.05	0.53
	Negative Emotional Expressiveness	0.25	0.45	0.04	0.57
	Hostility	−0.06	0.12	−0.04	0.62
Health Behaviors	Smoking	−0.87	0.94	−0.06	0.36
	Alcohol Consumption	0.05	0.05	0.05	0.38
	Recreational Physical Activity	−0.001	0.001	−0.03	0.57

**Table 3:** Psychosocial correlates of sleep duration among LGB women.

LG Women Hours of Sleep					
		B	SE	*b*	*p*
Model 1					
Psychosocial Characteristics	Social Strain	−0.03	0.02	−0.07	0.05
	Social Function	0.01	0.002	0.09	0.01
	Social Support	0.01	0.01	0.07	0.1
	Optimism	0.03	0.01	0.1	0.01
	Negative Emotional Expressiveness	−0.13	0.06	−0.08	0.03
	Hostility	0.04	0.02	0.09	0.02
Model 2					
Psychosocial Characteristics	Social Strain	−0.03	0.02	−0.06	0.23
	Social Function	0.01	0.002	0.11	0.02
	Social Support	0.01	0.01	0.08	0.12
	Optimism	0.03	0.02	0.09	0.06
	Negative Emotional Expressiveness	−0.17	0.07	−0.1	0.02
	Hostility	0.04	0.02	0.12	0.02
Health Behaviors	Smoking	−0.2	0.13	−0.07	0.13
	Alcohol Consumption	0.01	0.01	0.05	0.26
	Recreational Physical Activity	0.0003	0.0002	0.05	0.23
	Bisexual Women Hours of Sleep				
		B	SE	*b*	*p*
Model 1					
Psychosocial Characteristics	Social Strain	−0.04	0.02	−0.1	0.04
	Social Function	0.002	0.002	0.04	0.46
	Social Support	−0.005	0.007	−0.04	0.46
	Optimism	−0.001	0.01	−0.003	0.96
	Negative Emotional Expressiveness	0.05	0.08	0.03	0.55
	Hostility	−0.02	0.02	−0.05	0.36
Model 2					
Psychosocial Characteristics	Social Strain	−0.05	0.03	−0.13	0.05
	Social Function	0.001	0.003	0.01	0.81
	Social Support	−0.01	0.01	−0.06	0.37
	Optimism	0.004	0.02	0.01	0.83
	Negative Emotional Expressiveness	0.05	0.1	0.03	0.62
	Hostility	−0.02	0.03	−0.04	0.54
Health Behaviors	Smoking	−0.35	0.22	−0.1	0.11
	Alcohol Consumption	0.02	0.01	0.1	0.11
	Recreational Physical Activity	9.13 × 10^−6^	0.0004	0.002	0.98

## Data Availability

The data that support the findings of this study are available on request from the Women’s Health Initiative. The data are not publicly available due to protections for the privacy of research participants but can be requested from WHI.
